# Supplementary material for: Synchronous micromechanically resonant programmable photonic circuits
Source: Nat Commun. 2023 Nov 24;14:7716. doi: 10.1038/s41467-023-42866-3 (PMC10673894; doi:10.1038/s41467-023-42866-3)
Supplement: Supplementary file 1 — Supplementary Information [file 41467_2023_42866_MOESM1_ESM.pdf]

Synchronous micromechanically resonant programmable photonic circuits

Mark Dong,[1,2,*] Julia M. Boyle,[1] Kevin J. Palm,[1] Matthew Zimmermann,[1] Alex Witte,[1] Andrew J. Leenheer,[3] Daniel Dominguez,[3] Gerald Gilbert,[4,†] Matt Eichenfield,[3,5,†] and Dirk Englund[2,6,†]

[1]The MITRE Corporation, 202 Burlington Road, Bedford, Massachusetts 01730, USA

[2]Research Laboratory of Electronics, Massachusetts Institute of Technology, Cambridge, Massachusetts 02139, USA

[3]Sandia National Laboratories, P.O. Box 5800 Albuquerque, New Mexico 87185, USA

[4]The MITRE Corporation, 200 Forrestal Road, Princeton, New Jersey 08540, USA

[5]College of Optical Sciences, University of Arizona, Tucson, Arizona 85719, USA

[6]Brookhaven National Laboratory, 98 Rochester St, Upton, New York 11973, USA

[*]Corresponding Author: Mark Dong (mdong@mitre.org)

[†]Equally Contributing Authors: Gerald Gilbert (ggilbert@mitre.org), Matt Eichenfield (meichen@sandia.gov), Dirk Englund (englund@mit.edu)

1. Supplementary Methods

Experimental setup for characterizing the PIC. Supplementary Fig. 1 depicts the experimental setup for the resonant tree calibration and qualification. We used a Ti:Sapphire laser at 737 nm wavelength for the 1 x 8 photonic switch characterization. The light was coupled to the PIC from a fiber aligned to an on-chip grating. The input polarization was controlled with a half and quarter waveplate in order to maximize transmission into the TE mode of the SiN waveguide. The eight edge-coupled outputs of the switch were collected with a high-NA objective (0.9 NA) and refocused onto either an intensified charge coupled device (ICCD) or a 125 MHz photodiode. The collected light was filtered with a free-space linear polarizer before detection.

For electrical control, the photonic switch was cleaved and wire bonded to a custom printed circuit board (PCB). The cantilever phase shifters were electrically driven with two 625 MS/s arbitrary waveform generator (AWG) PXIe cards, each with four output channels with a voltage range of ± 2.5 V and 5x amplified on the PCB at a max slew rate of 8000 V·μs^{-1}. The cantilevers were operated in push pull configuration in order to maximize actuation. A laptop computer controlled all of the electrical equipment and sent programmed pulse sequences to the voltage controller, which then synchronously actuated all devices on the photonic switch. The computer then collected all data from the oscilloscope and camera for post-processing.

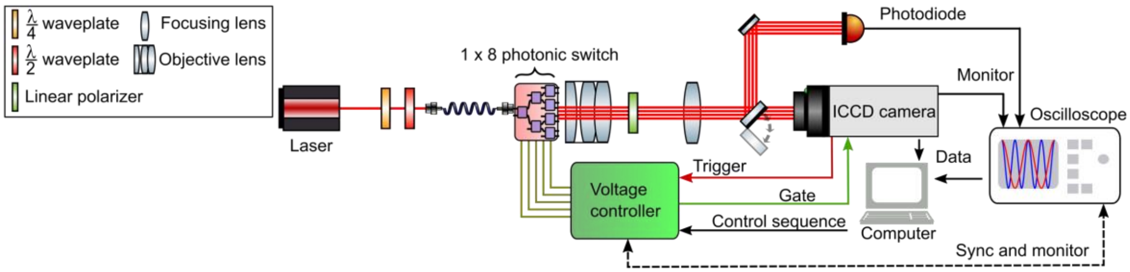

Supplementary Fig. 1: Experimental setup for characterization of the 1 x 8 photonic switch. Diagram of the components used in the experiment characterizing the photonic switch.

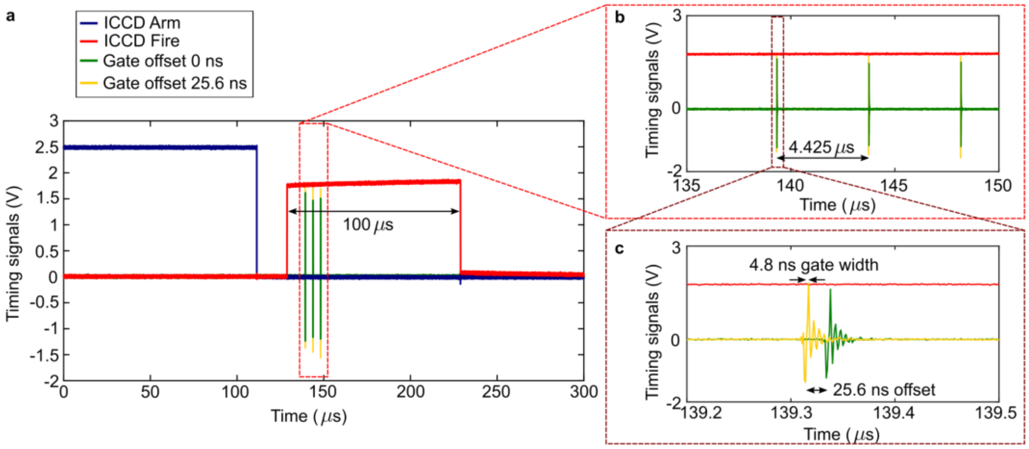

Supplementary Fig. 2: Timing signals for the ICCD. a) Monitored voltages from the ICCD's "arm" and "fire" channels, indicating when the camera starts the exposure. Two "gate" monitor signals from two separate image captures, indicating when the shutter has opened, are also plotted for comparison. b) Zoomed-in portion of the gate signals, showing three shutter opens per exposure. c) Further zoomed-in plot of the two gate monitor signals showing the widths and relative programmed delays between image captures.

Timing and triggering of voltage controller and ICCD. Supplementary Fig. 1 illustrates several timing signals between the computer, voltage controller, and ICCD camera. These signals from an exemplary exposure of the ICCD are plotted in Supplementary Fig. 2. In our high-speed imaging experiments, we first programmed all photonic control signals into the memory buffer of the voltage controllers. We then set the ICCD to start the exposure but with the shutter off initially. The "fire" monitor on the camera, which goes high when exposure is active (Supplementary Fig. 2a), was used to trigger the voltage controllers to execute the synchronized sinusoids to the PIC. In addition to controlling the PIC, the voltage controller also sent a synchronous "gate" signal to directly turn on the camera shutter at specific moments in time relative to the firing of the photonic switch. Only when the "gate" and "fire" signals are both on (TTL high) will the camera measure the optical outputs. We subsequently adjusted the timing offset of the gate signal to generate different images of the 8-channel output (Supplementary Fig. 2b, c). We see three distinct gate signals (4.8 ns width) per image capture to improve the signal-to-noise ratio, spaced at 50 times the fundamental frequency period (~4.425 μs). We note the spacing is limited by the ICCD direct-gate repetition limit of 200 kHz.

Device calibration and mechanical stability. We calibrated the photonic switch in sequence, storing all voltage values in a file that could be reloaded for each experiment. We first calibrated the DC voltages, specifically the 50:50 operating point, starting with MZIs in column 3 and proceeding to columns 2 and then 1. Next, we performed a small-signal frequency response measurement of the AC modulators (Fig. 4c-e in the main text) using a two-port vector network analyzer. We connected port 1 to the desired target device and port 2 to the photodiode output. By measuring the scattering parameters, specifically S21, we obtained the MZI response. From these plots, we then selected the base frequency (11.3 MHz in the main text) to optimize the overall modulation enhancement of all

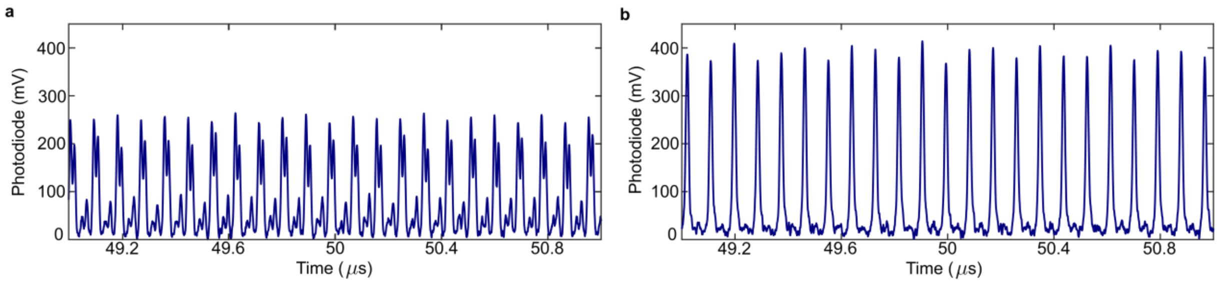

Supplementary Fig. 3: Phase alignment and compensation of MZIs. a) Time-resolved output of optical channel 6 before the MZI has undergone the calibration procedure. b) Time-resolved output of optical channel 6 after appropriate amplitude, offset, and phase values are implemented on the MZI.

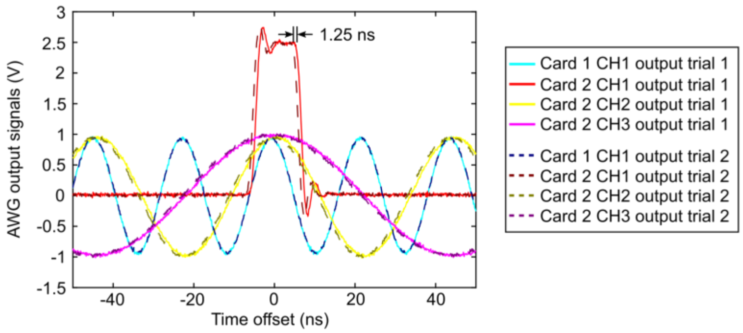

Supplementary Fig. 4: Timing differences between AWG driver cards. Plots of two separate scans of four channels from two separate AWG PXIe card. The plots of trials 1 and 2 show that, even though the two cards are synchronized and triggered together, the exact timing of the channels from different cards may vary by 1 sample or 1.25 ns.

devices. At 11.3 MHz, the modulation enhancement factors were 1.26, 3.69, 2.64, 6.01, 3.17, 1.50, and 1.93 for MZIs 11, 21, 22, 31, 32, 33, 34 respectively, averaging an overall 2.9x or 4.6 dB for the whole circuit. Lastly, we proceeded to optimize the output pulse sequence by adjusting the AC sinusoid amplitude and phase. Supplementary Fig. 3 shows the phase synchronization process, where we monitored the full switching sequence and adjusted the phases to achieve the desired switching order and compensate for the phase ϕ_c of each cantilever.

The timing between the output channels from separate AWG PXIe cards (M4x.6622-x4 Spectrum Instrumentation) used for electronic control is not deterministic. Even when synchronized and triggered, the timing delay will randomly vary by +/- one sample (1.25 ns for the 625 MHz sample rate driver), which is quite significant relative to our switching time (~11 ns). Supplementary Fig. 4 plots electronic output signals from four channels, one from card 1 and three from card 2, showing how the timing varies between successive fires. We see that all channels on the same card are always synchronized to each other, while channels from different cards may have a +/- one sample timing variation. This is a documented issue specific to the cards we used and is not a limitation of the PIC. Alternate electronic control systems with better timing options (e.g. AD9959 Analog Devices) would eliminate the synchronization uncertainty.

During the calibration procedure, we note that not all AC cantilevers were able to perfectly hit the on-off switching range. Specifically, the longer overhang cantilevers (all in column 3 MZIs) exhibited some mechanical instability when driven at high voltages. Supplementary Fig. 4 plots this phenomenon for two MZIs: a more stable MZI 34 and a less stable MZI 31. We applied digital signal processing techniques on the optical modulation resulting from each MZI and plotted the time-dependent amplitudes of all relevant harmonics. The dynamics of the less stable modulator (Supplementary Fig. 4d) suggests nonlinear mechanical coupling to lower frequency modes. To alleviate this effect, we applied lower voltage sinusoids (Fig. 5c in the main text) for better stability at the cost of lower switching fidelity. This nonlinear coupling does not occur on the shorter overhang cantilevers (MZIs in columns 1 and 2). We found that the excitation of the lower order modes can be reduced by changing the metal electrode patterning of the cantilevers – see the post-process trimming results in the Supplementary discussion below.

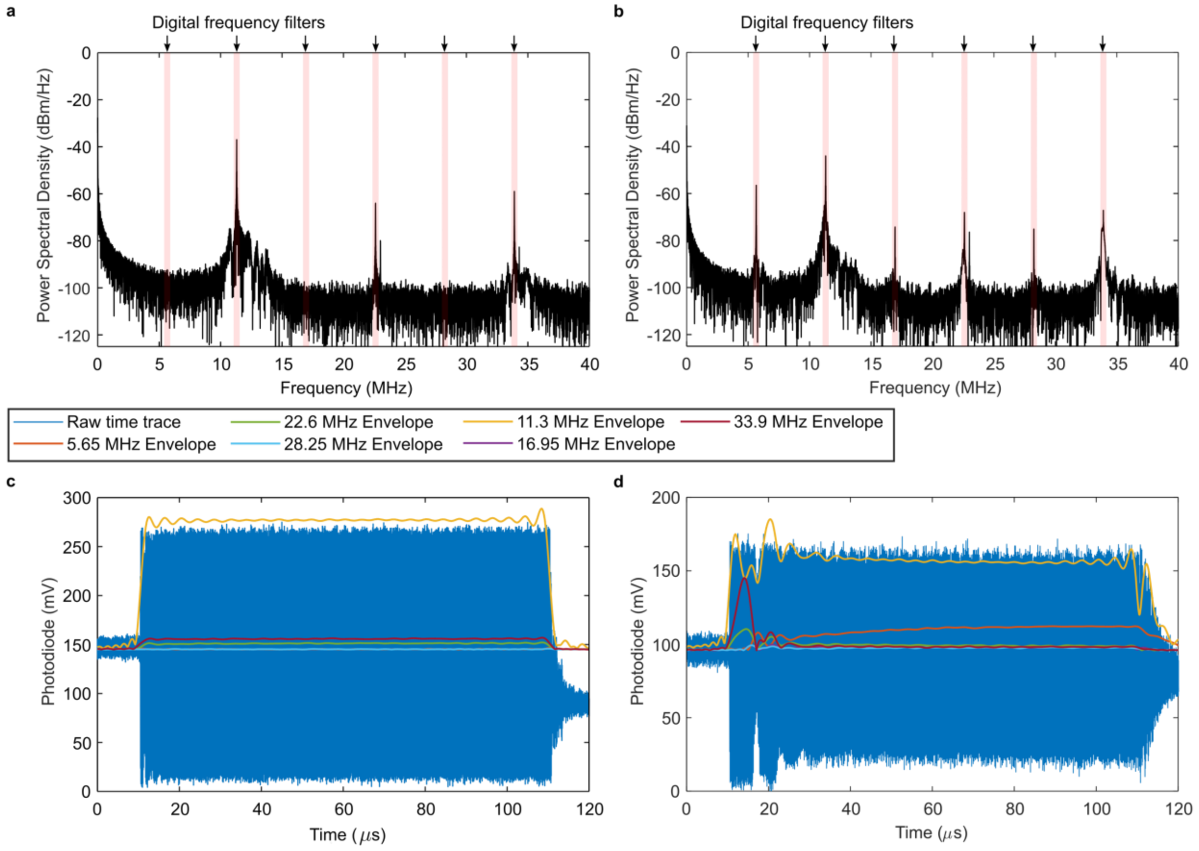

Supplementary Fig. 5: Mechanical harmonic generation and frequency drift in MZIs. Frequency and time-domain plots of the optical modulation when MZIs 31 (channel 2) and 34 (channel 7) were driven with a high-voltage amplitude (7.5 V) sinusoid of 11.3 MHz for approximately 100 µs. a) Fourier transform plots of the optical modulation for MZI 34 and b) MZI 31, showing various harmonics and the location of applied digital frequency filters. c) Time-domain plots of the optical modulation for MZI 34 and d) MZI 31, showing the amplitudes of the filtered harmonics. MZI 34 shows stable modulation of the different frequency components at the expected harmonics, while MZI 31 shows many more frequency components, including a subharmonic at 5.65 MHz, whose amplitudes are drifting over time.

2. Supplementary Discussion

Improvements in switching circuit design. While the basic design of the photonic switch (Fig. 3b in the main text) is efficient in terms of number of optical channels relative to the number of optical modulators, the pulses from each output overlap significantly in time domain (Fig. 3d and Fig. 5a in the main text). If the photonic switch is used

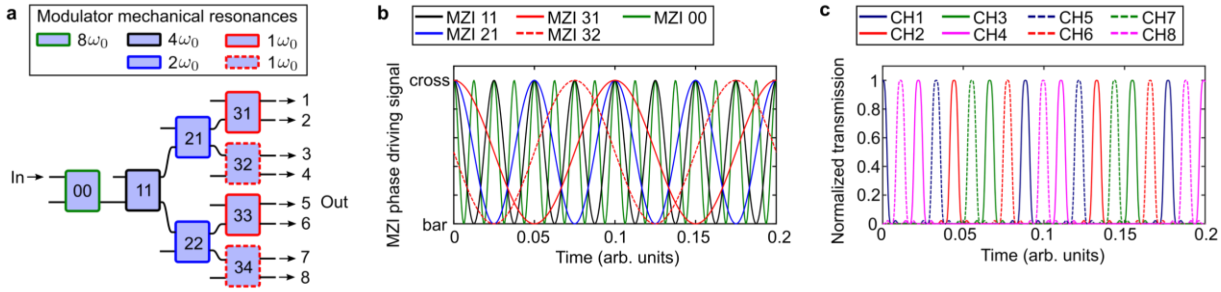

Supplementary Fig. 6: Design improvements to resonantly actuated 1 x *N* photonic switches. a) Schematic of a modified 1 x 8 photonic switch, showing a MZI 00 operating at 8 times the fundamental frequency added at the beginning of the switch's input. b) Plot of an example set of control signals for a subset of the MZIs, including the high-frequency MZI 00. c) Simulated optical outputs of the modified switch design, showing the effects of MZI 00 creating narrower pulses that have much less overlap in time compared to those without MZI 00.

primarily for pulse picking applications, the effects of the overlapping channels may not be as important. However, if the photonic switch is to be used for pulse carving applications, then an improved design is warranted. Supplementary Fig. 5 shows a modified photonic switch, with an additional high-frequency MZI at the input. This MZI, labeled 00, provides an additional frequency component to the overall switching signals, narrowing all pulses at the output (Supplementary Fig. 5c). We see that narrower pulses are possible with a trade-off of more modulators and control complexity.

Post-process trimming of cantilever modulators. The matching of resonances and actuation of the MEMS cantilever modulators may be significantly improved with a post-process trimming procedure[1]. We implemented a recipe for cutting the on-chip metal lines using a commercial laser system (Quantum Composers LS635B-S2). We applied the laser cutter in a specific pattern to the top metal (Supplementary Fig. 7a) as a way of tuning the mechanical resonance frequency. We chose this pattern to better match the electrodes to the actual shape of the simulated[2] mechanical resonance. The pre- and post-trimming results of a nominal 20 MHz cantilever are shown in Supplementary Fig. 7b. We see several important effects after trimming the aluminum top metal: 1) the peak mechanical resonance of interest has shifted by ~ 1.83 MHz; 2) the MZI response has increased significantly (from ~6.4 dB to ~9.8 dB enhancement) due to more efficient excitation of the higher-order modes while suppressing coupling to other lossy mechanical modes[1] and 3) the suppression of lower frequency modes results in a flatter bandwidth up until 10 MHz, where other modes start to appear. Effects 1) and 2) benefit our all-resonant architecture, while effect 3) could be positive or negative depending on how the trimmed mechanical modes interact. These results show how trimming techniques, with their flexibility and degrees of freedom, can help address some of the long-term engineering challenges of the resonant PIC architecture. We plan to study in more detail the laser trimming and mechanical effects in future work.

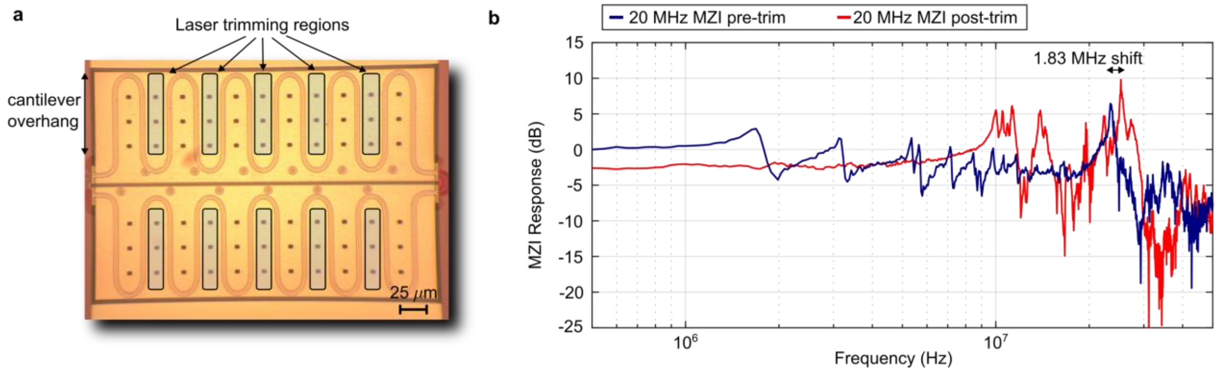

Supplementary Fig. 7: Post-process trimming of a cantilever modulator via laser metal cutting. a) Optical microscope image of a nominal 20 MHz cantilever showing the regions in which the laser trimming was applied. The laser cuts through the metal film in between the waveguides while avoiding the release holes. b) Measured small-signal response of the cantilever before and after trimming, showing shifting of the mechanical resonance. The modulation enhancement has also increased for the high frequency modes while suppressed for the lower frequency modes.

3. Supplementary References

1. Miller, J. M. L. *et al.* Effective quality factor tuning mechanisms in micromechanical resonators. *Appl. Phys. Rev.* **5**, 041307 (2018).

2. Dong, M. *et al.* Piezo-optomechanical cantilever modulators for VLSI visible photonics. *APL Photonics* **7**, 051304 (2022).
